# Evidence that osteogenic and neurogenic differentiation capability of epidural adipose tissue-derived stem cells was more pronounced than in subcutaneous cells

**DOI:** 10.3906/sag-2001-76

**Published:** 2020-12-17

**Authors:** Bilgehan SOLMAZ, Ali ŞAHİN, Taha KELEŞTEMUR, Ertuğrul KILIÇ, Erkan KAPTANOĞLU

**Affiliations:** 1 Department of Neurological Sciences, Marmara University, İstanbul Turkey; 2 Department of Neurosurgery, İstanbul Education Research Hospital, Ministry of Health, İstanbul Turkey; 3 Department of Physiology, İstanbul Medipol University, İstanbul Turkey; 4 Regenerative and Restorative Medical Research Center, İstanbul Medipol Universtiy, İstanbul Turkey; 5 Department of Neurosurgery, Başkent University, İstanbul Turkey

**Keywords:** Epidural adipose tissue, stem cell, differentiation, CD90, CD105

## Abstract

**Background/aim:**

The management of dura-related complications, such as the repairment of dural tears and reconstruction of large dural defects, remain the most challenging subjects of neurosurgery. Numerous surgical techniques and synthetic or autologous adjuvant materials have emerged as an adjunct to primary dural closure, which may result in further complications or side effects. Therefore, the subcutaneous autologous free adipose tissue graft has been recommended for the protection of the central nervous system and repairment of the meninges. In addition, human adipose tissue is also a source of multipotent stem cells. However, epidural adipose tissue seems more promising than subcutaneous because of the close location and intercellular communication with the spinal cord. Herein, it was aimed to define differentiation capability of both subcutaneous and epidural adipose tissue-derived stem cells (ASCs).

**Materials and methods:**

Human subcutaneous and epidural adipose tissue specimens were harvested from the primary incisional site and the lumbar epidural space during lumbar spinal surgery, and ASCs were isolated.

**Results:**

The results indicated that both types of ASCs expressed the cell surface markers, which are commonly expressed stem cells; however, epidural ASCs showed lower expression of CD90 than the subcutaneous ASCs. Moreover, it was demonstrated that the osteogenic and neurogenic differentiation capability of epidural adipose tissue-derived ASCs was more pronounced than that of the subcutaneous ASCs.

**Conclusion:**

Consequently, the impact of characterization of epidural ASCs will allow for a new understanding for dural as well as central nervous system healing and recovery after an injury.

## 1. Introduction

Despite the long history of spinal surgery, a primary watertight dural closure is still one of the most challenging subjects in neurosurgery. The cause of the current dural closure procedures may not be sufficient for the prevention of cerebro spinal fluid (CSF) leakage. Consequently, the treatment of leakage often requires extended postoperative hospitalization, placement of subarachnoid drainage, and surgical revision. These processes may frequently lead to further complications, such as deep vein thrombosis, pulmonary embolism, pneumonia, urinary tract infection, subdural or cerebellar hematomas, as well as increased medical expenses [1]. Numerous surgical techniques and synthetic or autologous adjuvant materials have emerged as an adjunct to primary dural closure, which may result in further complications or side effects [1,2]. Use of subcutaneous autologous free adipose tissue graft was previously recommended for the treatment of dural leakage, protection of neural tissue, and repair of the dura [3,4]. However, the exact cellular and molecular mechanisms underlying between the subcutaneous adipose tissue grafts and dural repair need to be investigated. Furthermore, the current knowledge about the dural healing mechanism is still largely unclear [5].

The mechanisms of tissue repair or tissue healing after an injury is complex with dynamic responses. Therefore, the types of tissues, severity of injury, and immune response are closely related in these processes. Recent studies have revealed that endogenous stem cells play a critical role in tissue healing with both complete regeneration and scar tissue formation [6]. Moreover, adult endogenous stem cells can also be recruited from distinct stem cell niches, such as adipose tissue, through signals from the extracellular matrix to support of healing process [7,8]. Consequently, the identification of stem cell sources at both the wound site and the surrounding tissues is required to develop new regenerative treatment strategies. Although, neural stem/progenitor cell activity has been demonstrated in meninges as a response to neural parenchymal injury [9], the roles of the spinal endogenous dural or meningeal stem cells are largely unknown.

The meninges are composed of pia, arachnoid, and dura mater, which are connective tissue coverings of the brain and spinal cord. Contrary to the cranial dura, the spinal dura is anchored within the vertebral canal by connective tissue strings, which is a series of pial thickenings and epidural ligaments that are associated with posterior longitudinal ligament, ligamentum flavum, and vertebral laminae [10]. Consequently, the spinal dura has a true potential space called an epidural space between the dura and vertebral canal, which extends from the base of the skull to the sacral hiatus. Embryologically, the epidural space is formed immediately after completion of the differentiation of the dura mater in the fetus at 13 weeks [11]. At the beginning, this space is filled with an undifferentiated loose mesenchymal tissue. Around week 32, differentiated adipocytes become apparent and then epidural adipose tissue constitutes of main component of this space in adults [11]. These mature adipocytes exhibit a regular hexagonal cell shape and as well as a unique honeycomb-patterned shape, which occurs between the dural sleeves that envelope the spinal nerve roots [12].

Because of this anatomical and ultrastructural close relationship, the epidural adipose tissue beyond the simple filler material was considered as a dynamic formation for the development and maintenance of spinal meninges. Furthermore, this unique alignment pattern of epidural adipocytes between the dural sleeves may play a critical role as a stem cell niche. And thus, a hypothesis was established that the epidural and also subcutaneous adipose tissue-derived stem cells (ASCs) might be an endogenous tool for the treatment of meningeal injury and central nervous system (CNS) repair. In order to prove this hypothesis, stem cells were isolated from the human epidural and subcutaneous adipose tissues and then the phenotypic properties and differentiation capacities of these cell populations were compared. These findings may encourage neurosurgeons to use adipose tissue for the prevention of CSF leakage. However, further clinical and experimental studies are necessary to elucidate the detailed role of adipose tissue grafting.

## 2. Material and methods

The Human Research Ethics Committee of Marmara University approved this study in accordance with the Declaration of Helsinki. Patients were asked to fill-in informed consent forms before their planned lumbar spinal surgical operation. The study participants comprised 3 patients aged between 35 and 50 years, who did not have any autoimmune or systemic diseases. Adipose tissue specimens were surgically obtained from the lumbar subcutaneous and epidural region during the lumbar spinal operation from the same individual.

### 2.1. Isolation of epidural and subcutaneous ASCs

Human ASCs were isolated and cultured, as described previously, with minor modifications [13]. Adipose tissue specimens were washed twice with phosphate-buffered saline (PBS) to remove extra blood, small vessels, and connective tissues. Subsequently, the cleaned adipose tissues were cut into very small pieces and put into two or three, 50-mL falcon tubes. Next, 0.075% type II collagenase was added to each falcon tube, which was followed by incubation at 37 °C for 60 min. The digestion process was stopped by adding an equal volume of PBS and the samples were then centrifuged at 600 *g* for 15 min at room temperature. The mature adipocytes, debris, and liquid portion were discarded, and the stromal vascular fraction (SVF) was precipitated. The SVF was resuspended in PBS and then centrifuged at 400 *g* for 10 min at 20 °C, and then transferred to culture flasks. Approximately 50 × 103 mixed SVF cells per cm2 were transferred into each flask. Next, Dulbecco’s modified Eagle’s medium (DMEM), supplemented with 10% fetal bovine serum (FBS), was added to each culture flask and placed into the incubator at 37 °C, with 5% CO2 and humidified air. After 48 h of incubation, the cells were washed to remove the culture medium and non-adherent cells. Moreover, the culture medium was renewed with fresh medium every 72 h. At 85%–90% confluence, the adipose tissue-derived mesenchymal stem cells (AT-MSCs) were harvested using the enzymatic method and the cells were subcultured while the cell count number, cell viability, and purity were evaluated using Trypan blue (Invitrogen, Carlsbad, CA, USA) and a hemocytometer.

### 2.2. Phenotypic characterization of epidural and subcutaneous AT-MSCs

ASC surface markers were analyzed using the flow cytometry criteria of the MSCs, as defined previously [14]. Cells were characterized using cell surface markers via flow cytometry analysis. Briefly, 1 × 105 cultured ASCs at 3rd subculture which were resuspended in buffer [3% bovine serum albumin (BSA)], in PBS, and then appropriate fluorescein isothiocyanate (FITC), u2922 (PE), and PECy5 conjugated primary antibodies for 30 min at 4 °C. CD34-PE and CD45-PECy5 were used as the negative control, and CD90-FITC and CD105-FITC were used as the positive control and isotypic control and afterwards, the labeled AT-MSCs were analyzed on a Guava easyCyte flow cytometry instrument (Luminex Corp., Austin, TX, USA).

### 2.3. In vitro differentiation assay

Adipogenic, osteogenic, and neuronal differentiation assays were performed according to the methods described previously, with minor modifications [15].

### 2.4. Adipogenic differentiation

Cells were incubated in adipogenic differentiation medium that was constructed by supplementing complete DMEM with 50 µg/mL of indomethacin, 10-7 M of dexamethasone, and 50 µg/mL of ascorbate-sodium 2-phosphate (all from Sigma-Aldrich, St. Louis, MO, USA). The culture medium was renewed every 72 h. After 14 days, the differentiated cells were fixed with 10% formalin for 1 h and washed with 60% isopropanol and stained with oil red O for 10 min. The oil red O-stained cells were solubilized in 100% isopropanol.

### 2.5. Osteogenic differentiation

Cells were incubated in osteogenic differentiation medium (DMEM supplemented with 10 mM of ß-glycerol phosphate, 50 µg/mL of ascorbate sodium 2-phosphate, and 10-7 M of dexamethasone, 1% antibiotic/antimycotic (Sigma-Aldrich) for 21 days. After differentiation of the cells, they were fixed using ethanol for 10 min and stained with Alizarin Red S.

### 2.6. Neuronal differentiation

To induce neuronal differentiation, the cells were cultured for 5 days in differentiation medium (DMEM supplemented with 0.5 m of Misobutylmethylxanthine, 10 ng/mL of brain-derived neurotrophic factor, 10 ng/mL of epidermal growth factor, 10 ng/mL of basic-fibroblast growth factor, 20% neural stem cell proliferation supplement). Neuronal differentiation was evaluated by morphological changes and neural marker expression such as Nestin, glial fibrillary acidic protein (GFAP), and 2’,3’-Cyclic-nucleotide 3’-phosphodiesterase (CNPase). After neural induction, the cells were fixed with 4% paraformaldehyde for 15 min at room temperature, incubated for 30 min in blocking solution (5% (w/v) BSA, 0.6% (v/v) Triton X-100 in PBS), and then incubated with each primary antibody anti-Nestin (A11861, ABclonal), anti-GFAP (A10873, ABclonal) and anti-CNPase (A1018, ABclonal) at room temperature for 3 h. Subsequently, the cells were rinsed with washing buffer and incubated at room temperature for 1 h with appropriate fluorochrome-conjugated secondary antibodies. Microscopy analysis was performed with laser confocal microscopy (Zeiss LSM 700, Oberkochen, Germany).

### 2.7. Western blot analysis

Total protein from both cell types was extracted using cell lysis buffer (Cell Signaling Technology, Inc., Danvers, MA, USA). The protein concentrations of the supernatants were determined via bicinchoninic acid assay. First, 30 µg of the protein samples were separated with 10%–12% SDS-PAGE gels and transferred to nitrocellulose membranes. Membranes were incubated with primary antibodies against Nestin, GFAP, and CNPase. Following the use of horseradish peroxidase-conjugated secondary antibodies and a chemiluminescence kit (Cell Signaling Technology, Inc.), the blots were quantified and normalized with glyceraldehyde 3-phosphate dehydrogenase.

### 2.8. Statistical analysis

Statistical analysis was performed using Prism 4 (GraphPad Software, San Diego, CA, USA) software. For determination of the statistical significance of the differences, the unpaired t test was performed followed by the 2-tail test, and P < 0.05 was considered as statistically significant.

## 3. Results

### 3.1. Morphological evaluation of epidural and subcutaneous fat tissue-derived stem cells

**Figure 1:**
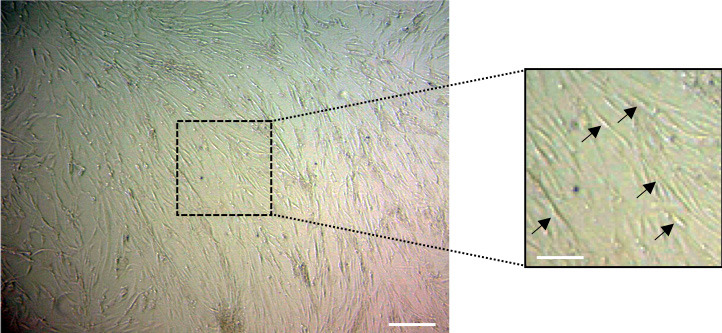

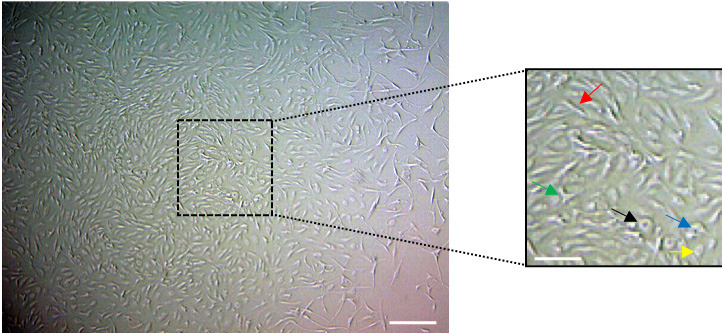
Morphology of ASCs isolated from lumbar subcutaneous and epidural fat tissues cultured in vitro. (A) After being plated in a petri dish for 24 h, the primary adherent cells, which were isolated from subcutaneous tissue and grew into spindle-shaped (black arrows), appeared to adopt a more uniform fibroblast-like shape. (B) After being plated in a petri dish for 24 h, the primary adherent cells, which were isolated from epidural tissue, grew into heterogenous morphologies, which were spindle-shaped (red arrow) with different directionality and irregularity, and also triangular (blue arrow), polygonal (green arrow), small ovoid (yellow arrow), and round (black arrow).

Cells from subcutaneous fat tissue were observed to have mesenchymal stem cell morphology, such as a spindle shape, with thin and long cell processes at first passage (P0), as seen in Figure 1a. Cells from epidural fat tissue were observed to have heterogenous morphology, such as triangular, polygonal, small ovoid, and spindle-shaped at first passage (P0), as seen in Figure 1b.

### 3.2. Phenotypic characterization of epidural and subcutaneous fat tissue-derived stem cells

**Figure 2:**
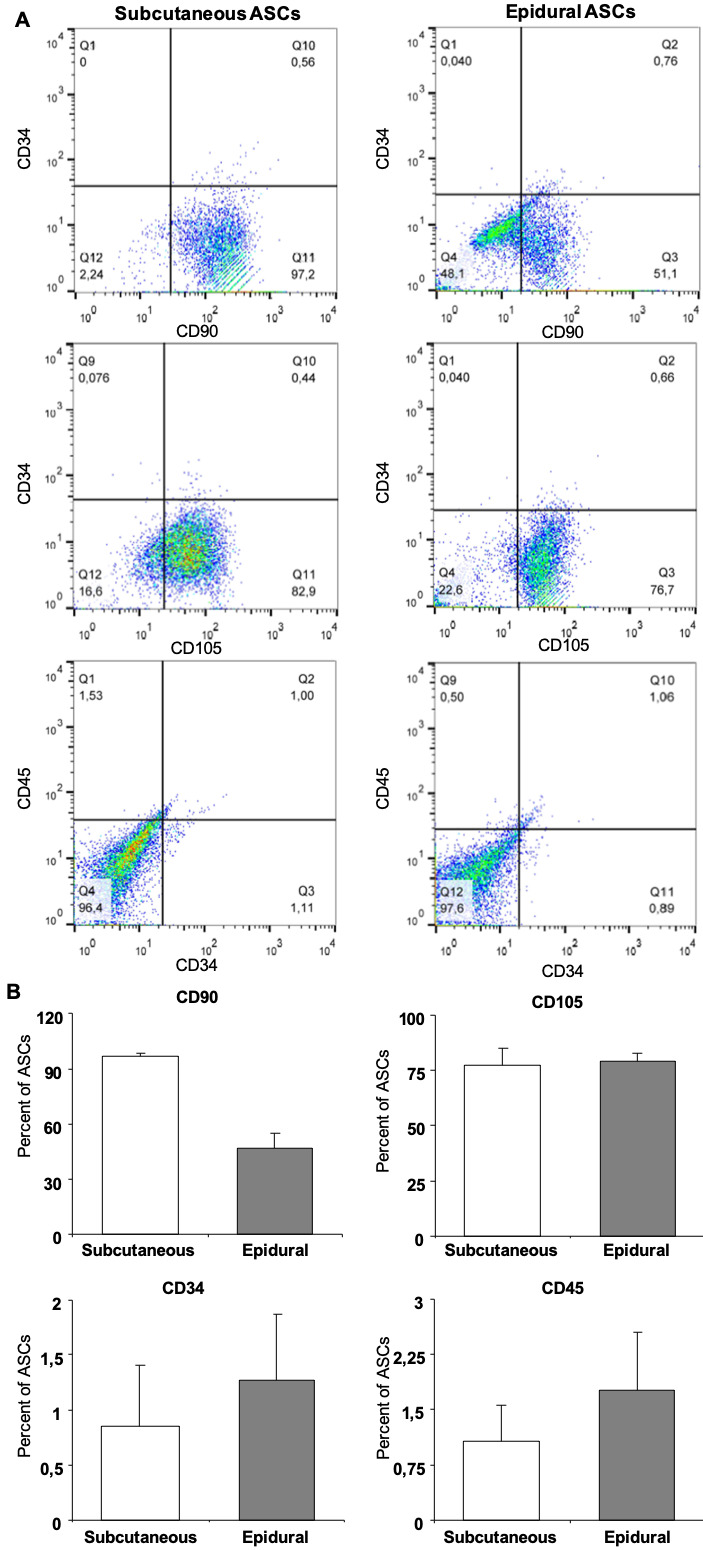
Cell surface marker expression for subcutaneous and epidural ASCs (A). Flow cytometry analysis results showed that both the subcutaneous and epidural ASCs expressed a significantly low percentage of CD34 and CD45 (B). The typical surface markers of stem cells, CD90 and CD105, were expressed in both types of ASCs (2B); however, the epidural ASCs exhibited significantly lower CD90 expression than the subcutaneous ASCs.

Plastic adherent stem cell colonies were observed in all of the donor samples within the first days of cultivation (Figure 2a). Flow cytometry analysis revealed that both the subcutaneous and epidural ASCs expressed a significantly low percentage of CD34 and CD45, with the average percentages as 0.85 ± 0.56 and 1.07 ± 0.49, and 1.27 ± 0.60 and 1.76 ± 0.78, respectively, as seen in Figure 2b. The typical surface markers of stem cells, CD90 and CD105, were expressed in both types of ASCs; however, the epidural ASCs exhibited significantly lower CD90 expression than the subcutaneous ASC average percentages, as 47.07 ± 8.25 and 96.71 ± 1.95, respectively.

### 3.3. Expression of neural and glial proteins by the undifferentiated epidural and subcutaneous ASCs

**Figure 3:**
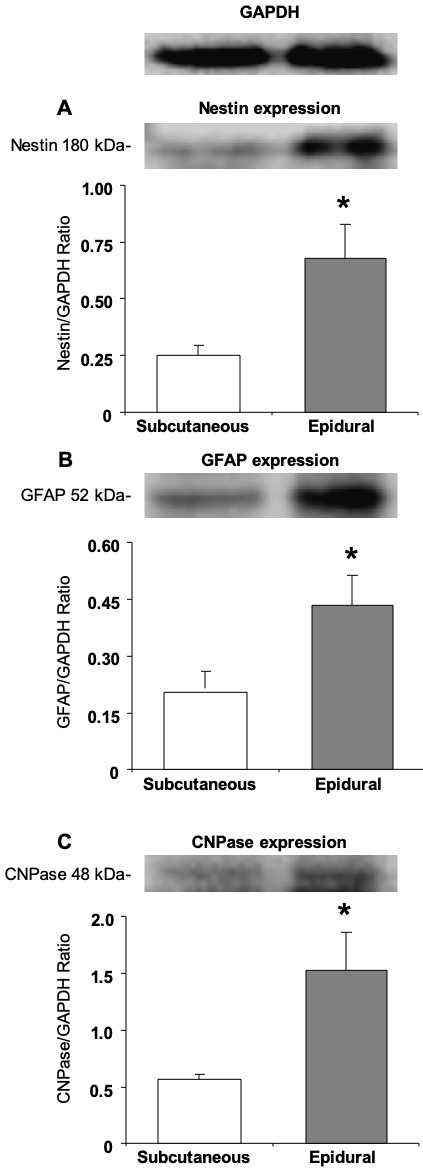
Western blot analysis in order to detect neural cell proteins Nestin, GFAP, and CNPase of the subcutaneous and epidural ASCs before neural induction, and at the fifth passage, both subcutaneous and epidural adipose tissue-derived cultures expressed these proteins and it seemed with a significant difference. The expression of Nestin (*P < 0.05), GFAP (*P < 0.05), and CNPase (*P < 0.05) genes had a significant difference in the epidural ASCs.

Also investigated was the protein expression of Nestin (an intermediate filament protein originally described in neural stem cells), GFAP (an intermediate filament protein that is expressed by numerous cell types of the CNS, including astrocytes and ependymal cells), and CNPase (a myelin- associated enzyme) in the undifferentiated epidural and subcutaneous ASCs. Both undifferentiated groups showed expressions of the protein Nestin, GFAP, and CNPase. There was a significant difference between the subcutaneous and epidural ASCs for Nestin, GFAP and CNPase (P < 0.05), as seen in Figure 3.

### 3.4. Comparison of the adipogenic differentiation of the subcutaneous and epidural ASCs

**Figure 4:**
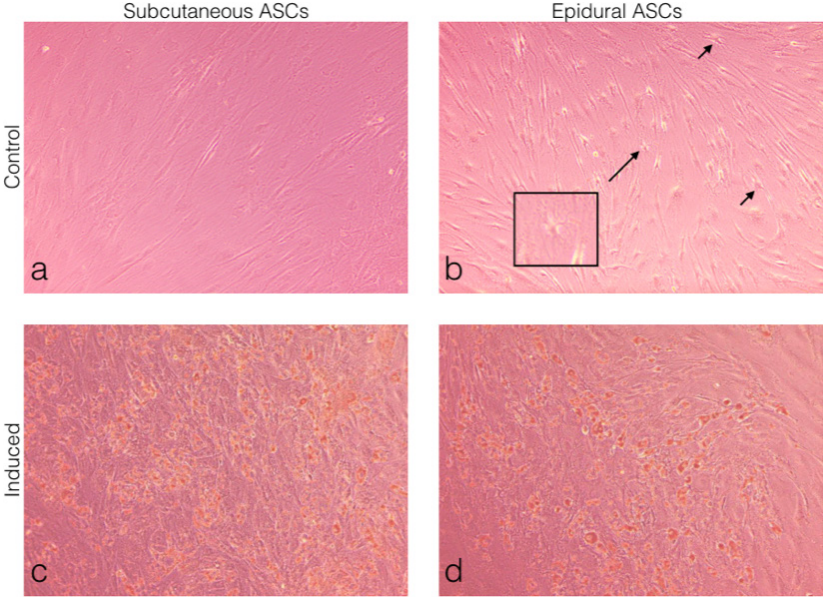
Morphological observations of the ASCs under light microscopy. (a) Subcutaneous ASCs cultured in normal growth medium for 14 days. (b) Epidural ASCs cultured in normal growth medium for 14 days. (c) Subcutaneous ASCs cultured in adipogenic medium for 14 days. (d) Epidural ASCs cultured in adipogenic medium for 14 days. Oil red O staining showed the formation of lipid droplets in both induced groups (c and d). Differentiated cells were observed in the control of the epidural SCs. The arrows indicate star-shaped cells, with the box showing a further magnified view (b).

Subcutaneous and epidural ASCs were induced to a standard adipogenic induction culture to examine the adipogenic differentiation and then compared to the noninduced cells or the control, as seen in Figures 4a and 4b. The oil red O staining results showed small lipid droplets in the cytoplasm, which were morphologically similar in both the subcutaneous and epidural ASCs, as seen in Figures 4c and 4d. No oil red O-positive cells were observed in the control specimens. Furthermore, as shown in Figure 4b, differentiated cells were observed in the noninduced controls of the epidural ASCs.

### 3.5. Comparison of the osteogenic differentiation of the subcutaneous and epidural ASCs.

**Figure 5:**
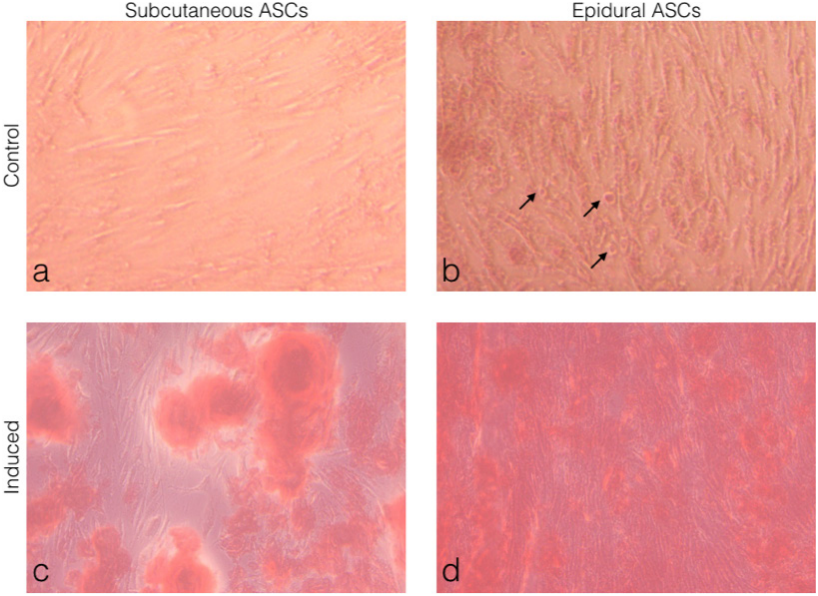
Morphological observations of the ASCs under light microscopy. (a) Subcutaneous ASCs cultured in normal growth medium for 21 days. (b) Epidural ASCs cultured in normal growth medium for 21 days. (c) Subcutaneous ASCs cultured in adipogenic medium for 21 days. (d) Epidural ASCs cultured in adipogenic medium for 21 days. Alizarin red S staining showed the deposit of calcium crystals in both induced groups. (b) The arrows indicate round differentiated cells in the control of the epidural ASCs.

Subcutaneous and epidural ASCs were induced into a standard osteogenic induction culture to examine the osteogenic differentiation and when compared to the noninduced cells or the control, as seen in Figures 5a and 5b. The alizarin red S staining results showed a deposit of calcium crystals, which was higher in the epidural ASCs when compared with the subcutaneous ASCs, as seen in (Figures 5c and 5d). No alizarin red S-positive cells were observed in the control cultures. Furthermore, as shown in Figure 5b, differentiated cells were observed in the noninduced controls of the epidural ASCs.

### 3.6. Comparison of the neurogenic differentiation of the subcutaneous and epidural ASCs.

**Figure 6:**
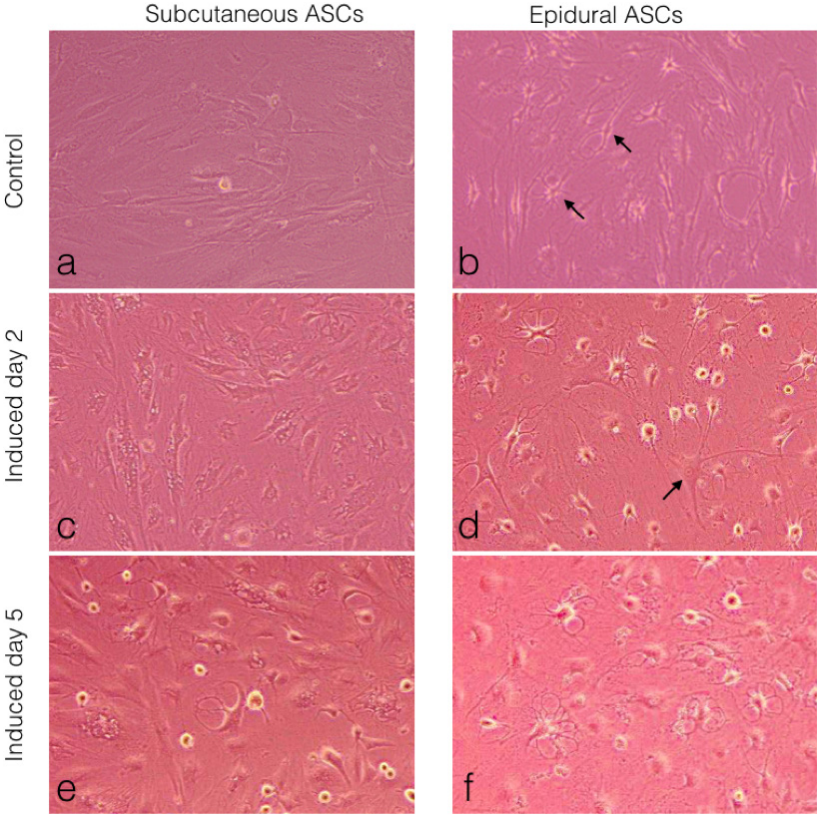
Morphological observations of the ASCs under light microscopy. (a) Subcutaneous ASCs cultured in normal growth medium for 5 days. (b) Epidural ASCs cultured in normal growth medium for 5 days. Arrows indicate neuroglia and neuron like cells. (c) Subcutaneous ASCs cultured in neurogenic medium for 2 days. (d) Epidural ASCs cultured in neurogenic medium for 2 days. The arrow indicates a neuron-like cell with a cell body, containing the nucleus and the surrounding cytoplasm, with several short and long radiating branches, similar to dendrites and an axon. (e) Subcutaneous ASCs cultured in neurogenic medium for 5 days. (f) Epidural ASCs cultured in neurogenic medium for 5 days.

Subcutaneous and epidural ASCs were induced into a standard neurogenic induction culture to examine the neurogenic differentiation and then compared to the noninduced cells or the control, as seen in (Figures 6a and 6b). Following 2 days of neural induction, the epidural ASCs displayed neuronal or glial-like cells in their morphology, as seen in Figure 6d, when compared with the subcutaneous ASCs (Figure 6c). After 5 days, both groups of cells displayed changes in their cellular morphology, including shrinkage of the cytoplasm, the formation of axons, and dendrite-like cytoplasmic projections (Figures 6e and 6f). However, this morphological differentiation was more pronounced in the epidural ASCs. Furthermore, as shown in Figure 6b, neuroglia-like cells were observed in the noninduced controls of the epidural ASCs

### 3.7. Comparative morphological analysis by immunocytochemistry after neural induction of the subcutaneous and epidural ASCs

**Figure 7:**
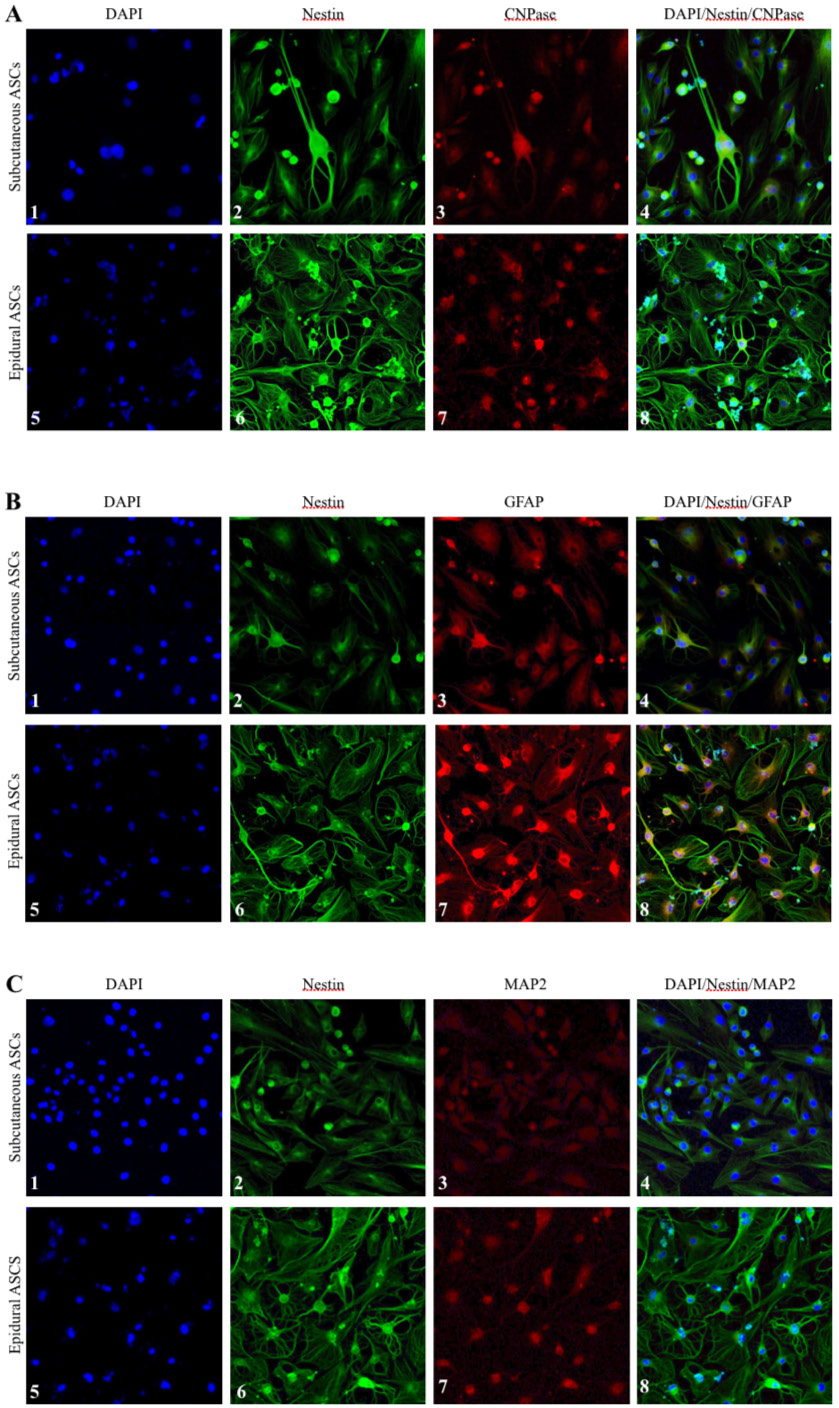
Morphological observations of the neurogenic differentiation of the subcutaneous and epidural ASCs under laser confocal microscopy. Fluorescence micrographs of the 4′,6-diamidino-2-phenylindole-stained cell nuclei (blue), as seen in panels A, B, and C. Nestin marker expression (green) was seen in all of the panels. Expression of CNPase marker (red), as seen in panels A3 and A7. Expression of GFAP marker (red), as seen in panels B3 and B7. Expression of MAP2 marker (red), as seen in panel C3 and C7.

To investigate the expression of Nestin, CNPase, GFAP, and MAP-2 markers in the neural differentiated cells derived from the subcutaneous and epidural ASCs, immunocytochemistry staining was performed, as seen in Figure 7. The results of the immunofluorescence microscopy analysis confirmed the expression of these markers on day 5 of neural induction in both groups of cells. However, there was a remarkable morphological difference in the neurogenic differentiated cells between the subcutaneous and epidural ASCs in all of the samples, which requires further comparison studies of both the subcutaneous and epidural ASCs for the elucidation of their roles in meningeal and parenchymal repair after an CNS injury.

## 4. Discussion

Adipose tissue is connective tissue that is composed mainly of fat-storing cells (adipocytes), and other cell types (i.e. endothelial cells, fibroblasts, neuronal cells, and leukocytes) within a structural network of fibers [16,17]. Adipose tissue resides in specific anatomic locations, which are also called fat depots. These depots are categorized as intraabdominal or visceral fat deposits, mainly located in the upper and lower portions of the body, subcutaneously [18]. The macroscopic appearance of these depots resembles each another, which is called white adipose tissue (WAT). The other specialized fat deposit is called brown adipose tissue (BAT), which has a dark pigment due to the high density of mitochondria in its cytochromes. In the current study, both samples from the lumbar-epidural and lumbar-spinal subcutaneous fat tissues were WATs, according to this traditional classification [19]. The primary function of WATs is to store and release fatty acid molecules in response to changes in the energy balance. Furthermore, WATs have different roles, including immune, endocrine, mechanical, and thermal functions [20]. In addition to these major WAT deposits, there are tissue-associated WAT deposits that are often small in size and broadly distributed all around the body. These depots are closely associated with the adjacent anatomic structures [21]. In consideration of their anatomical and ultrastructural close relationship with the dura mater and epidural fat tissue, it can be supposed that a discrete tissue-associated adipose deposit. Recent studies have shown that tissue accumulation WAT deposits have diverse functions, such as the modulation of tissue growth and tissue repair or regeneration process, especially in stem cell-rich tissue (i.e. bone marrow, skin and mammary gland) [22]. The current knowledge about epidural fat tissue is only related with biomechanical support for its surrounding tissues, such as dura mater and nerve roots against the movement of the vertebral column [19]. However, due to advances in the identification of its distinct functions, novel therapeutic approaches are likely to emerge that are associated with spinal pathologies.

Consequently, the first step to understanding the functions of tissue-associated WAT deposits is to understand their developmental origins. However, adipose tissue has a heterogenous mixture of cells, known as the SVF, except in mature adipocytes, as previously mentioned. Therefore, it is hard to say that there is only one source for the developmental origin for each adipose deposit [23]. In addition, the SVF contains an abundant population of multipotent stem cells [24,25]. These cells are called ASCs, and are adherent to plastic culture flasks, can be expanded in vitro, and have the ability to differentiate into ectodermal, endodermal, and mesodermal lineages [26]. Basically, ASCs are mesenchymal stem cells (MSCs) that are obtained from fat tissue. For this reason, these are supposed to express MSC surface markers, such as CD90, CD105, CD73, CD44, and CD166 and also the negative expression of the hematopoietic surface markers CD45, CD34, and HLA-DR. However, these markers do not consistently express all of the characteristics of MSCs, and the profile expression may vary with the culture time and its environment [27].

In accordance with the current results, ASCs contain uniform characteristic markers, positive for CD90 and 105, and negative for CD34 and CD45. However, the study results revealed that the expression of CD90 was a lower percentage in epidural ASCs (47.07%) when compared with subcutaneous ASCs (96.7%). The cluster differentiation 90 (CD90), which is also known as Thy-1, is a 25–37 KDa glycosylphosphatidylinositol-anchored glycoprotein expressed on MSCs [28]. Despite an increasing number of studies, from wound healing to neuritis outgrowth modulation, in various cell types related with the function of CD90 expression, its function in stem cell biology remains unclear. In addition, the molecular pathways that can be transformed into osteogenesis have not been completely elucidated. However, a reduction in CD90 expression leads to a more efficient osteogenic differentiation [29–31]. In the present study, the reduction of CD 90 expression may have been related to the more pronounced formation of the osteogenic matrix after osteogenic induction in the epidural ASCs when compared to the subcutaneous ASCs.

Another characteristic feature of undifferentiated ASCs is their typically bipolar or multipolar elongated shape, called fibroblast-like cells [32]. The study results revealed that the isolated epidural ASCs exhibited heterogeneous shapes apart from the fibroblast-like morphology, on the contrary to the subcutaneous ASCs (Figure 1). The cell shape was related with, as well as internal factors, biochemical and genetic materials, active properties balanced by external forces such as compression, tension and shearing [33]. In this sense, mature epidural adipocytes that are spread around the dura, and also within the dural sleeves, may have a regular hexagonal shape that is directly related to the balance of the physical forces exerted on the cell surface. These external forces can be attributed to cerebrospinal fluid pressure fluctuation in the dural sac and spinal epidural space pressure. Interestingly, recent studies have shown that these external physical factors affect the shape of stem cells as well as their fate of differentiation. For instance, the increased gravity caused the changing the shape of the MSCs and improved the differentiation of the cells into osteoblasts [34]. Consequently, mechanical forces within the epidural space and the regular hexagonal shape of the mature epidural adipocytes may have been the cause of the change in physical shape of the epidural ASCs, as was observed herein.

Furthermore, differentiated cells, like neuroglia cells, were observed in both the adipogenic and neurogenic control cultures of the epidural ASCs, without any neural induction (Figures 3B and 5b). To date, only a single study has reported that the spontaneous formation of neural-like cells could occur without any growth factors in ASCs [35]. For this reason, the expression level of neural differentiation markers between the epidural and subcutaneous ASCs in the undifferentiated culture medium was compared, and statistically high levels of neural stem cell marker, Nestin, glial marker, GFAP, and oligodendrocyte and Schwann cells marker, CNPase, in epidural ASCs were observed (Figure 2). It is well known that stem cell morphologies and their gene or protein expressions can be different, depending on their source tissues. Furthermore, undifferentiated ASCs can express native immature neural proteins, but after neuronal differentiation, these expressions will be reduced [33,36,37]. However, in the current study, it was observed that the neurogenic differentiation was more pronounced and began earlier in the epidural ASCs when compared with the subcutaneous ASCs, after neural induction (Figures 5d and 5f). Compatible with this, the immunoreactivities for Nestin, GFAP, CNPase, and MAP-2 of in vitro neurogenic differentiated epidural ASCs were visualized. Interestingly, star-shaped differentiated stem cells were observed in almost every microscopic field in all of the samples of the epidural ASCs (Figures 6b, 6d, and 6f). These findings suggested that epidural adipose tissue may be a stem cell niche for the CNS. After all, these isolated stem cells from both epidural and subcutaneous fat tissues should be able to transform into the mature cell types, which have characteristic morphologies and specialized functions compatible with the source tissue. In this sense, there was no morphological difference between the adipogenic differentiation potentials of the epidural and subcutaneous ASCs (Figures 4C and 4D). However, the heterogeneity of the stem cell shape and neurogenic differentiation ability may indicate the developmental origin of epidural ASCs. Actually, recent studies have suggested different developmental origins, aside from mesoderm, which has been the generally accepted origin for ASCs (or MSCs) [38–40]. Specifically, lineage-tracing studies have revealed that a small number of ASCs have originated from the neural crest at some certain areas in the cephalic region [41,42]. These distinct minor populations of stem cells exhibited markers for both neural crest progenitors and preadipocytes [43]. Although the exact functions of these cells remains unclear, interestingly, they displayed bipolar and multipolar cell morphologies that were different then the characteristic fibroblast-like shape [43]. Consequently, in light of the fact that the neural crest was conclusively determined as a developmental origin of spinal meninges in mammals, it was considered herein that the neural crest-derived stem cells may also contribute to the formation of human epidural fat tissue. In conclusion, we have demonstrated that osteogenic and neurogenic differentiation capability of epidural ASCs were more pronounced than subcutaneous ASCs. However, further studies toward the elucidation of the mechanisms of osteogenic and neurogenic differentiation need to be planned.
